# Mechanisms Governing Metabolic Heterogeneity in Breast Cancer and Other Tumors

**DOI:** 10.3389/fonc.2021.700629

**Published:** 2021-09-23

**Authors:** Sayani Patra, Naveed Elahi, Aaron Armorer, Swathi Arunachalam, Joshua Omala, Iman Hamid, Anthony W. Ashton, David Joyce, Xuanmao Jiao, Richard G. Pestell

**Affiliations:** ^1^ Pensylvania Cancer and Regenerative Medicine Research Center, Baruch S. Blumberg Institute, Wynnewood, PA, United States; ^2^ Xavier University School of Medicine at Aruba, Oranjestad, Aruba; ^3^ Program in Cardiovascular Medicine, Lankenau Institute for Medical Research, Wynnewood, PA, United States; ^4^ Medical School, Faculty of Health and Medical Sciences, The University of Western Australia, Crawley, WA, Australia; ^5^ Cancer Center, Wistar Institute, Philadelphia, PA, United States

**Keywords:** breast cancer, metabolism, Warburg effect, aerobic glycolysis, reverse Warburg effect, epigenetics, PPAR-γ, Cyclin D1

## Abstract

Reprogramming of metabolic priorities promotes tumor progression. Our understanding of the Warburg effect, based on studies of cultured cancer cells, has evolved to a more complex understanding of tumor metabolism within an ecosystem that provides and catabolizes diverse nutrients provided by the local tumor microenvironment. Recent studies have illustrated that heterogeneous metabolic changes occur at the level of tumor type, tumor subtype, within the tumor itself, and within the tumor microenvironment. Thus, altered metabolism occurs in cancer cells and in the tumor microenvironment (fibroblasts, immune cells and fat cells). Herein we describe how these growth advantages are obtained through either “convergent” genetic changes, in which common metabolic properties are induced as a final common pathway induced by diverse oncogene factors, or “divergent” genetic changes, in which distinct factors lead to subtype-selective phenotypes and thereby tumor heterogeneity. Metabolic heterogeneity allows subtyping of cancers and further metabolic heterogeneity occurs within the same tumor mass thought of as “microenvironmental metabolic nesting”. Furthermore, recent findings show that mutations of metabolic genes arise in the majority of tumors providing an opportunity for the development of more robust metabolic models of an individual patient’s tumor. The focus of this review is on the mechanisms governing this metabolic heterogeneity in breast cancer.

## Introduction

Breast Cancer. Breast cancer is the most common non-dermatological malignancy in women representing approximately one third of all malignancies diagnosed in US women (1, 2). In approximately 10% of cases breast cancers are associated with gene mutations inherited from one relative. Almost 50% of breast cancer cases occur in less developed countries with incidence rates ranging from 19 per 100,000 women in Eastern Africa to 90 per 100,000 women in Western Europe. Efforts to provide more precise therapies to patients with breast cancer has led to subclassification using the coding genome, the non-coding genome or more recently, metabolic subtypes.

Precision medicine approaches have identified genetic subtypes of BCa based initially on the coding genome (3). At least five distinct coding genome molecular subtypes are recognized including luminal A, luminal B, human epidermal growth factor receptor 2 (HER2)-enriched, basal-like, and claudin-low and normal-like (4, 5). Triple negative breast cancer (TNBC), which lacks estrogen receptor-α (ERα), progesterone receptor (PR) and HER2, characteristically includes mutations of DNA damage repair (6), altered PD-L1 expression (7) and increased expression of the G protein coupled receptor CCR5 (8, 9). Breast cancer has also been characterized based on the non-coding genome (10, 11). Altered expression of miRNA was observed in breast cancer (12). In subsequent studies hierarchical clustering of human breast cancers defined four distinct miRNA clusters (G1-G4) associated with distinguishable relapse-free survival by Kaplan-Meier analysis (10). These studies defined a cyclin D1-regulated miRNA signature which included several oncomirs, that was conserved in multiple breast cancer cell lines, and was associated with the G2 tumor miRNA cluster, ERα^+^ status, better outcome and activation of the Wnt pathway (10). Of interest these studies showed that the coding and non-coding genome for any given tumor were discordant within breast cancer subtypes. In recent studies metabolic subtyping of breast cancer has revealed distinguishable characteristics. Triple negative breast cancer (TNBC), for example, expresses low levels of glutamine synthetase (GLUL, glutamate-ammonia ligase). Withdrawing glutamine suppresses growth of the basal and claudin low triple negative tumors BCa tumor subtype (13). In contrast, luminal tumor cells express GLUL and are resistant to glutamine deprivation (14). The glutaminase isozyme GLS2, is upregulated and essential in luminal-subtype breast tumors (15).

Currently, therapies for breast cancer rely on surgical, radiotherapeutic, chemotherapeutic and biological therapeutic approaches (16). Despite these advances in medicine, 30% of patients relapse and develop a metastatic cancer (17). Patients with triple negative breast cancer (ERα Negative, HER2 Negative, PR Negative) have a poor outcome and require additional therapy. Historically targeting of the coding genome has improved mortality rates in breast cancer patients, and *in vitro* pre-clinical studies have shown promise in targeting the non-coding genome (18–20). The identification of metabolic dependencies specific to the cancer *vs.* normal cells therefore represents an important new opportunity for therapeutic intervention. In this regard the xCT antiporter, which is expressed on 1/3 of triple negative tumors *in vivo*, is essential to support environmental cystine acquisition (13). Inhibition of the xCT antiporter with the anti-inflammatory Sulfasalazine decreased tumor growth (13). Targeting GLS1/GLS2 with the small-molecule inhibitor 968 reduced tumor growth in luminal breast cancer (15).

**Figure 1:**
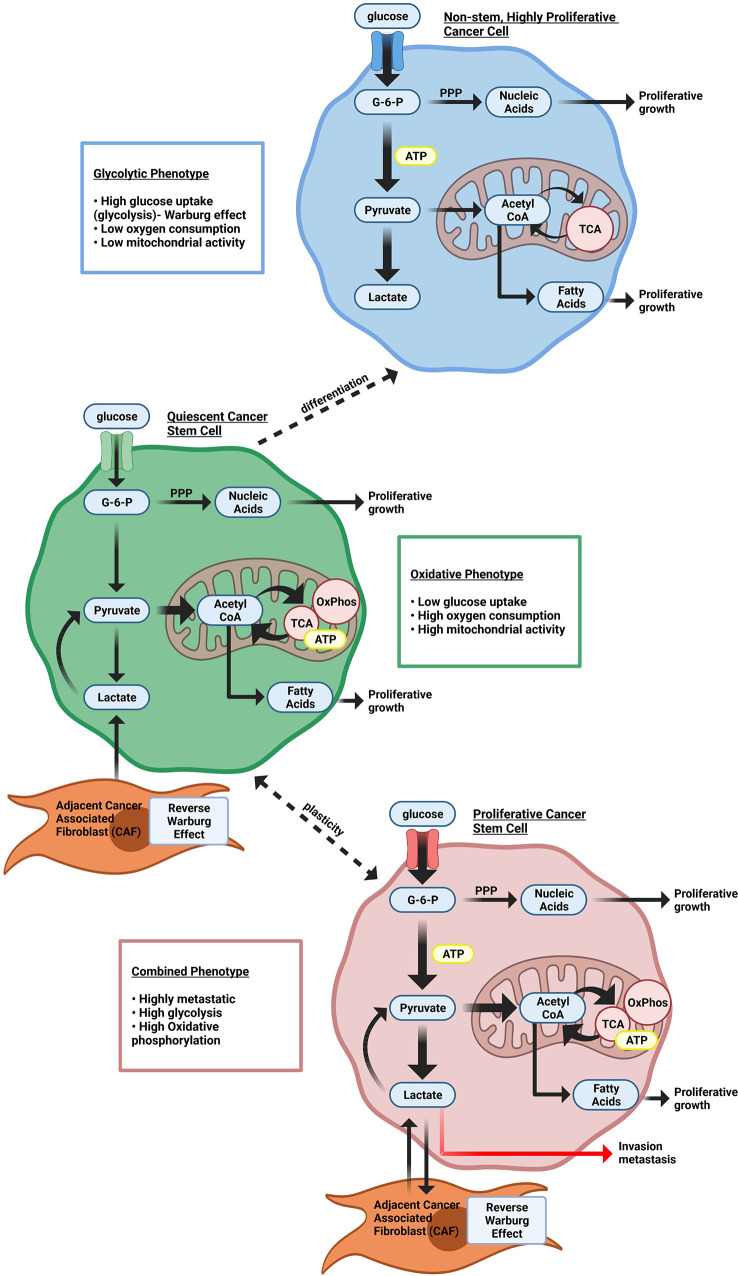
Heterogeneous cellular energy metabolism within breast cancer cells. Highly proliferating breast cancer cells rely on glycolysis to provide cellular energy. Quiescent breast cancer stem cells use more oxidative phosphorylation (OxPhos) instead of glycolysis to generate ATP. Proliferating cancer stem cells rely on both glycolysis and Oxphos. Both quiescent and proliferating cancer stem cells can use catabolites from cancer-associated stromal cells through the Reverse Warburg effect.

Tumor metabolism. The abnormalities associated with tumor metabolism have been recently reviewed (21–23). In contrast to normal differentiated cells, which rely primarily on mitochondrial oxidative phosphorylation to generate the energy needed for cellular processes, most cancer cells instead rely substantially on cytosolic aerobic glycolysis, a phenomenon termed “the Warburg effect” (**Figure 1**) (24, 25). In Warburg’s view, so central were metabolic changes to the cancer phenotype that he opined, “*From this point of view, mutation and carcinogenic agent are not alternatives, but empty words, unless metabolically specified*” (24).

As the analytical tools used to interrogate the characteristics of tumor metabolism have evolved, it has become increasingly clear that metabolic adaptations of tumors are highly heterogeneous (26–30). Historically the understanding of cancer metabolism was drawn from principles of “convergent” metabolic phenotypes, properties that are governed by diverse factors shared among diverse tumor types. As the resolution of investigative tools has evolved, evidence for “divergent” metabolic pathways has provided compelling evidence for breast cancer metabolic heterogeneity.

Convergent properties include the principal that cancer cells have evolved multiple distinct mechanisms in order to provide metabolic substrates for proliferation within the tumor microenvironment (25). Like all living cells cancer cells need ATP, together with carbon intermediates for the synthesis of DNA, proteins and lipids. Cancer cells augment the procurement of nutrients, scavenge nutrients from alternative sources (alternative substrates), reprogram metabolic process needed for growth, and upregulate the apparatus for processing the nutrients into energy and structural intermediates for replication, growth, and invasion. Cancer-associated metabolic changes have been usefully categorized as: (i) deregulated uptake of glucose and amino acids, (ii) opportunistic nutrient acquisition from both intracellular and environmental sources, (iii) use of glycolysis/TCA cycle intermediates for biosynthesis and NADPH production, (iv) increased demand for nitrogen and means to satisfy it, (v) alterations in metabolite-driven gene regulation, and (vi) metabolic interactions with the tumor microenvironment. Tumors vary in the degree to which they deploy these individual changes (31). Thus, in addition to changes in glucose uptake, increased levels of methionine, glutamine, cystine, tryptophan, tyrosine, and other amino acids have been noted in breast cancer (32–36). Cancer cells with upregulation of amino acid metabolism stimulate increased transport of amino acids into the cell. The increased consumption of amino acids and overexpression of amino acid transporters (L-type amino acid transporter 1) during breast cancer progression, has led to an interest in radiolabeled amino acids imaging agents (37).

“Divergent” properties derive from distinct genetic or epigenetic alterations with a tumor which govern distinct molecular subsets of genes that in turn alter cellular metabolism thereby contributing to metabolic heterogeneity. Distinct oncogenotypes have been characterized in a variety of cancers. IDH1 and IDH2 mutations give rise to accumulation of (R)-2HG (38, 39). In lung cancer distinct somatic mutations (TP53, KRAS, BRAF, NF1, EGFR, KEAP1) appear to be each sufficient to regulate tumor metabolism (26–28). Although less well characterized in breast cancer, genetic alterations that occur in breast cancer (cyclin D1 overexpression (40, 41) and epigenetic changes [FBP1 (42), the Jumonji-domain histone demethylase (JHDM)3C (43)], are sufficient to induces metabolic changes reflected by the Warburg effect.

## “Convergent” Genetic Properties Driving The Warburg Effect

Warburg observed that cancer cells primarily supply energy from glucose through avid glycolysis, even in aerobic conditions where more efficient mitochondrial oxidative phosphorylation (OXPHOS) was potentially available. Per molecule of glucose, glycolysis followed by OXPHOS generates up to 18 times more adenosine 5´-triphosphate (ATP) than glycolysis alone (44). The Warburg effect is pervasive among cancer cells of many but not all cancer types, For example the Warburg effect is not prominent in early prostate cancer (45, 46) and is found in only one of the metabolic subtypes of glioblastoma multiforme (21). Furthermore, the Warburg effects occurs in a heterogeneous manner within tumors, generating an intratumoral “nesting phenomenon”. Aerobic cells proliferate best when they are clustered with some glycolytic cells (44, 47, 48).

Several convergent genetic processes further drive the Warburg effect in tumors. Oncogenic mutations, tumor suppressor deletions and overexpression of collaborative oncogenes contributes to the tumor metabolic shift as these genes govern expression of glycolytic enzymes. In this regard *c-myc*, k-Ras, mutant p53, cyclin D1 and the β-catenin/TCF signaling pathway augment the Warburg effect (41, 49–52). Hypoxia-inducible factor-1α (HIF1α), which normally contributes to regulation of glycolysis in hypoxia, is also more protected from degradation in some cancer types.

A transition towards aerobic glycolysis is available to normal cells during proliferation (53). In tumor cells that activate the Warburg effect, several metabolic consequences occur. Cancer cells accelerate aerobic glycolysis partly through regulatory processes that are general to proliferating cells (54). Normally, glycolysis proceeds at a rate that reflects negative feedback control of intracellular ATP and NAD^+^/NADH homeostasis. There may be an advantage for an energetically active cancer cell in the speed of ATP production in glycolysis. ATP can be rapidly synthesized by glycolysis, up to 100 times faster than OXPHOs (55). Cancer cells, with diminished OXPHOS-generated ATP and efficient export of NADH reducing equivalents as lactate, maintain a permissive intracellular environment for dysregulated glycolysis. The diversion of pyruvate away from acetyl CoA production to lactate, simultaneously regenerates NAD^+^ and deprives the mitochondrial electron transport chain of NADH for ATP synthesis.

Aerobic glycolysis also confers an advantage to cancer cells by generation of macromolecules to increase the cellular biomass (53). Rapid proliferation of cancer cells needs to be sustained by increased macromolecular biosynthesis. As well as its role in providing substrates for mitochondrial OXPHOS, glycolysis intermediates supply the pentose phosphate pathway, support NADPH generation, contribute one-carbon species into the one-carbon cycle and make acetyl CoA available for lipid synthesis (53). Glucose-6-phosphate dehydrogenase action on the first product of glycolysis, glucose-6-phosphate, initiates the pentose phosphate pathway. Products of the pentose phosphate pathway include ribose-5-phosphate for nucleotide synthesis and NADPH. NADPH, an essential intracellular reductant that is consumed in numerous lipid, amino acid and nucleotide anabolic pathways, is created in both the pentose phosphate pathway and in one-carbon cycle reactions (56).

Later steps in glycolysis yield fructose-6-phosphate, which may proceed to hexosamine synthesis and dihydroacetone phosphate, which is a substrate for glycerol-3-phosphate, and thence lipid, synthesis. Enhanced expression of 3-phosphoglycerate dehydrogenase has been described in breast cancer cells (57). In glycolysis, 3-phosphoglycerate is diverted out of the pathway under the action of 3-phosphoglycerate dehydrogenase. This is a quantitatively important source of substrates for the one-carbon cycle, enabling production of glycine, serine and thence S-adenosylmethionine and one-carbon derivatives of tetrahydrofolic acid (58). These are essential for the synthesis of purine and pyrimidine bases for nucleotides and many other biosynthetic processes. The glycolysis product, pyruvate, is available for acetyl CoA generation and thereby lipogenesis (53, 59).

Increased *lactate production* from aerobic glycolysis leading to cellular acidification and results in lactate efflux from cells to maintain cellular pH. Lactate efflux results in an acidic extracellular tumor microenvironment, which promotes angiogenesis (60) increasing HIF1α stabilization, promoting VEGF production from cancer associated macrophages (61), enhancing hyaluronic acid production from fibroblasts (62), inducing extracellular matrix degrading cathepsins and matrix metaloproteases (63, 64), and augmenting endothelial cell PI3K and NFκb signaling (65) thereby promoting vasculogenesis.

## Epigenetic Changes in Tumors that Promote the Warburg Effect

**Figure 2:**
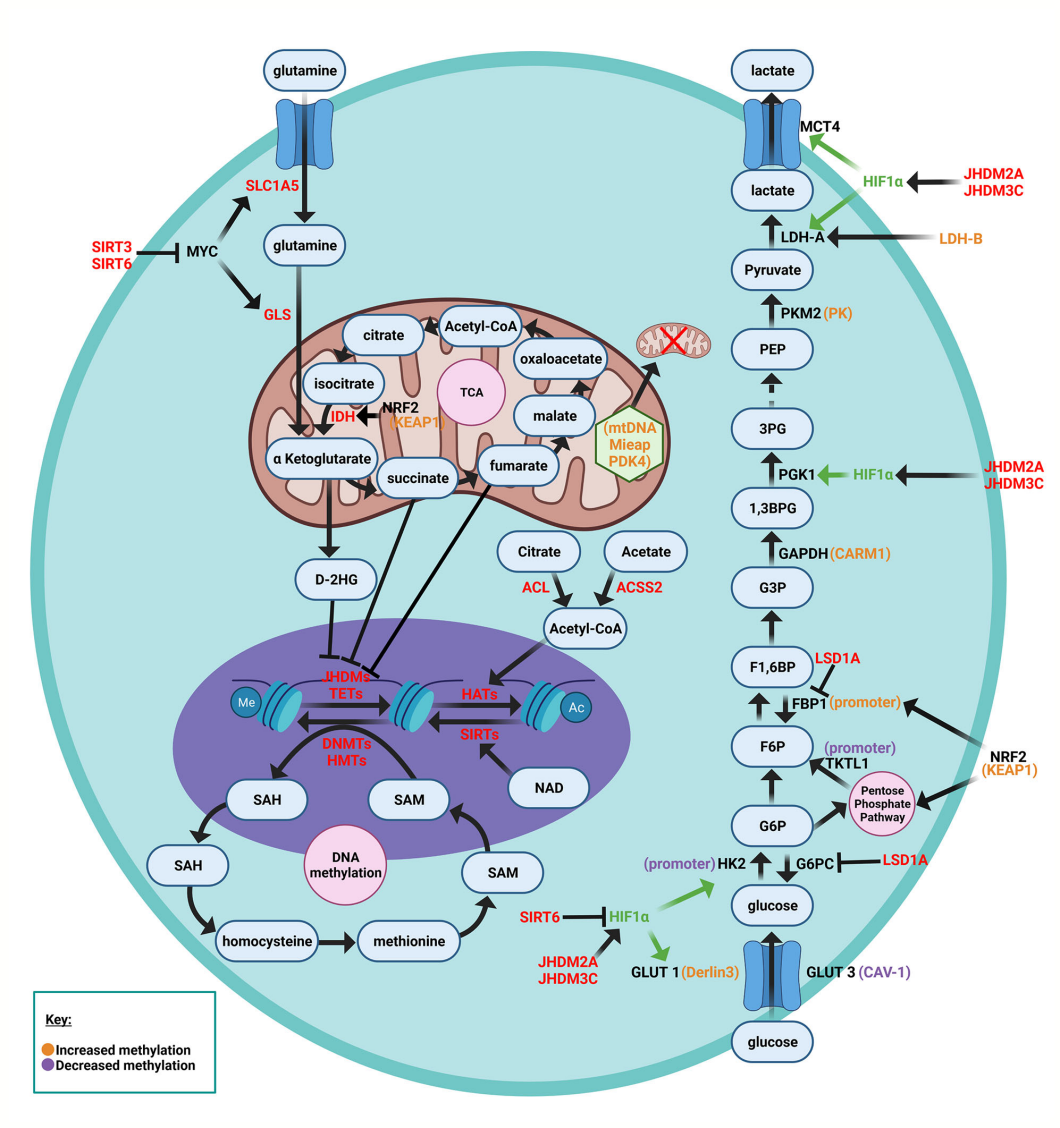
Schematic representation of metabolic nodes governed by epigenetic modification. Metabolic enzymes and nutrient transporters are governed by modification by DNA methylation and histone modifications. Bidirectional feedback occurs as the metabolic substrates generated in turn regulate epigenetic modifications. α-ketoglutarate is a co-substrate for JHDM (The Jumonji-domain histone demethylase) histone demethylase and the TET family methylcytosine dioxygenases, thereby governing demethylation of histone and other proteins, and DNA. Fumarate, succinate and 2-HG compete with a ketoglutarate. HIF1α (hypoxia inducible factor 1α), KEAP1 (Kelch-like ECH-associated protein 1), NRF2 (nuclear factor erythroid 2-related factor 2), FBP1 (fructose-1,6-bisphosphate isoform 1), TKTL1 (transketolase (TKT) like-1 gene), SAM (S-adenosyl methionine), TCA (tricarboxylic acid), The abundance of GLUT3 and GLUT1 are regulated by CAV-1 and Derlin3 respectively.

Epigenetic modifications in breast cancer cells can alter metabolism in a particular tumor, thereby contributing to divergent heterogeneous metabolic changes. Epigenetic changes, which are inheritable, reversible changes in selective gene expression that occur without any alterations to the DNA sequence itself, include DNA methylation, histone modifications, and RNA-mediated gene silencing by non-coding RNAs such as miRNA. DNA methylation has been described of specific key components in glycolytic pathways, glycolysis bypass pathways (i.e. gluconeogenesis and pentose phosphate pathway), as well as mitochondrial and oxygen sensing pathways (66). Furthermore many of the intermediates of cellular metabolic pathways participate in the chemical modifications that epigenetically modify DNA and histones (67). A synopsis of these epigenetic changes that have been described in tumors, shown in **Figure 2**, underscore the growing evidence for heterogeneous drivers of altered tumor metabolism. In addition to epigenetic changes within the tumors, epigenetic changes also occur in the tumor stroma. Distinct epigenetic alterations occur in epithelial and myoepithelial cells, and stromal fibroblasts occur in a tumor stage- and cell type-specific manner (68). Based on unsupervised analysis three methylation patterns of breast cancer (luminal A, luminal B and basal-like molecular subtypes) were identified, whereas HER2-enriched and normal-like subtypes were distributed among the three groups (69). The luminal B were most methylated and basal-like tumors least frequently methylated. BRCA2-mutated tumors were highly methylated. A large fraction of genes reported as having subtype-specific expression patterns might be regulated through methylation (69).

### Epigenetic Modification of the Tumor

Within the tumor, DNA methylation affects key glycolytic components, including glucose transporters (GLUT1, GLUT3), lactate dehydrogenase genes (*LDH-A, LDH-B*), the hexokinase 2 isoform (HK2), glyceraldehyde-3-phosphate dehydrogenase (GAPDH), and the pyruvate kinase (PK) isoform M2 (PKM2), each of which contribute to the Warburg effect (66). DNA hypermethylation-mediated inactivation of the *Derlin-3* gene, which normally contributes to GLUT1 degradation, leads to increased GLUT1 expression (70). Increased GLUT3 expression is regulated by methylation (71). DNA hypermethylation of the *LDH-B* gene in breast cancer (which interconverts lactate and pyruvate), increases the LDH-A to LDH-B ratio (72). Increased LDH-A mediated conversion of pyruvate into lactate promotes aerobic glycolysis (73). LDH-A activity is crucial to the Warburg effect since it oxidizes NADH and regenerates NAD^+^, without which aerobic glycolysis could not continue (74). Aerobic glycolysis is induced by upregulation of the hexokinase isoform HK2 *via* hypomethylation of its promoter (75) and hypomethylation within intron 1 of the pyruvate kinase (*PK* gene) (76). GAPDH upregulation, *via* coactivator-associated arginine methyltransferase 1 (CARM1)-mediated methylation also enhances aerobic glycolysis (77). The pyruvate kinase (PK) isoform M2 undergoes DNA methylation at exon 10 of the PK gene, correlating with increased PKM2 expression in breast cancer cells (78–80). Binding of Brother of Regulator of Imprinted Sites (BORIS) to the alternative exon 10 is thereby enhanced. Inhibiting DNA methylation, depleting BORIS or eliminating the BORIS binding site, switched splicing toward generating the normal PKM1 isoform. Loss of BORIS also suppresses the Warburg effect and growth of breast cancer cells (79).

DNA methylation of mitochondrial components, such as mitochondrial DNA (mtDNA), the mitochondrial quality control protein Mieap, and pyruvate dehydrogenase (PDH) kinase 4 (PDK4), causes mitochondrial dysfunction in cancer cells, triggering the Warburg effect (81). Methylation of mtDNA specifically causes dysfunction of oxidative phosphorylation, which promotes aerobic glycolysis as the primary method for rapid ATP synthesis in cancer cells (82). Mieap normally functions to induce intramitochondrial recruitment of lysosome-like organelles in order to eliminate oxidized mitochondrial proteins while maintaining mitochondrial structural integrity. Methylation of the *Mieap* promoter reduces Mieap abundance, leading to ROS accumulation and mitochondrial destruction (83–85).

DNA methylation changes affect activity of nuclear factor erythroid 2-related factor 2 (NRF2), which in turn regulates expression of transketolase (TKT) like-1 gene (TKT L1), and the fructose-1,6-bisphosphate isoform 1 (FBP1) (66). Methylation in the KEAP1 promoter reduces KEAP1 expression, and thereby abrogates NRF2 degradation (86–88). NRF2 is a transcriptional activator of genes in the pentose phosphate pathway [G6PDH, 6-phosphogluconate dehydrogenase (6PGD), transketolase (TKT), transaldolase and IDH (89)].

In basal-like breast cancer (42) and other cancer types (66), reduced Fructose-1,6-bisphosphatase (FBP1) expression, is due to *FBP1* promoter methylation (42). Reduced FBP activity increases glycolysis, enhancing glucose uptake and reducing OXPHOS. The FBP1’s substrate, fructose-1,6-bisphosphate, is an allosteric activator of PKM2, providing a link between reduced FBP1 activity and the Warburg effect (90).

DNA methylation regulates HIF1 abundance and key components in the oxygen sensing pathway (91), including the tumor suppressor proteins WW-domain containing oxidoreductase (Wwox), carboxy-terminal domain 4 (CITED4), the LIM domain containing protein (LIMD1), and von Hippel-Lindau (VHL) (66). In breast cancer GLUT1 expression inversely correlated with Wwox (92). DNA methylation regulates *Wwox* expression, which modulates glucose metabolism (93). Under aerobic conditions, loss of Wwox reduces mitochondrial respiration and activates glycolytic gene expression, thereby inducing the Warburg effect (94). This is linked to accumulation of Wwox associates with HIF1-α, facilitating hydroxylation by prolyl hydroxylase 2 (PHD2) (92). CITED4 inhibits the HIF complex. Hypermethylation of the *CITED4* promoter, reduces CITED4 expression in breast cancer, thereby increasing the expression of HIF and its target genes (95).

In melanoma cells and head and neck cancer cells, DNA hypomethylation of the *TKT L1* gene promoter increases TKT L1 expression and activity, promoting HIF1-α accumulation and stability (96) and inducing the Warburg effect (96, 97). LIMD1 acts as a scaffold protein to bind PHD2 and VHL, which degrade HIF by ubiquitination (98) and increased methylation of *LIMD1* and *VHL* are associated with upregulation of HIF1-α in cervical cancer (99).

### Epigenetic Modification of the Tumor Microenvironment

Epigenetic modification of the tumor microenvironment also contributes to tumor metabolic heterogeneity. Epigenetic reprogramming in CAFs are biomarkers for cancer progression and promote cancer epithelial progression *via* paracrine signaling (100). Multiple epigenetic mechanisms, including DNA methylation, histone modification, and chromatin remodeling, together shape and reprogram the phenotypes of CAFs during tumorigenesis (101). Altered DNA methylation status of genes occurs in CAFs isolated from breast cancer tissues (68) and the prostate (102). CAF-secreted factors and stromal content of breast tumors regulated specific genes characterized by a DNA methylation pattern: hypermethylation at transcription start site and shore regions (103). CAFs from localized prostate cancer display distinct genome-wide changes in DNA methylation, significantly at enhancers and promoters, compared to nonmalignant prostate fibroblasts (NPFs) (104). In pancreatic cancer CAFs adopt unique DNA methylation and expression patterns upon interaction with PDA tumor cells (105). Fibroblasts can be reprogrammed to adopt a pro-invasive phenotype by leukemia inducible factor (LIF), which induced methylation through DNMT3B of the promoter region in the protein phosphatase regulator Src homology 2 domain-containing protein tyrosine phosphatase 1 (*Shp-1*) gene (106).

Immune cells, including tumor-associated macrophages (TAMs), participate in breast cancer onset and progression and contribute to the TME metabolic ecosystem to enhance tumor growth. TAMs generally show increased aerobic glycolysis but may use OXPHOS to generate energy. Bidirectional metabolic feedback occurs between macrophages and breast cancer cells in which M2 like macrophages induce sodium/glucose cotransporter 1 (SGLT1) in breast cancer cells and SGLT1 enhances lactic acid secretion to promote M2 macrophage polarization (107) CCL5 activates the CCR5 receptor, which participates in metastasis of breast (9, 108) and other cancers  (109–111). Lactate induces the TAM phenotype, inducing CCL5 expression which promotes breast cancer cellular EMT and aerobic glycolysis *via* AMPK (112). Human mesenchymal stem cells (MSCs) induce the DNA methylation of the *IL1A* and *IL1B* genes when co-cultured with pancreatic ductal adenocarcinoma cells (PDAC) (113). The process of T-cell exhaustion is also controlled by epigenetic regulation and enrichment of T lymphocytes within the TME is a prerequisite for successful cancer immunotherapy (101).

Metabolic substrates from the tumor microenvironment in turn regulate methylation of stromal CAFs (114). The loss of cytosine methylation in de-novo generated CAFs is associated with the induction of inflammatory transcripts. Lactate produced by tumor cells leads to increased production of alpha-ketoglutarate (αKG) within mesenchymal stem cells (MSCs). In turn, αKG mediates activation of the demethylase TET enzyme leading to decreased cytosine methylation and increased hydroxymethylation during *de novo* differentiation of MSCs to CAF. Thus, in PDAC, a tumor-mediated lactate flux is associated with widespread epigenomic reprogramming that is seen during CAF formation.

## The Demand for Glutamine

**Figure 3:**
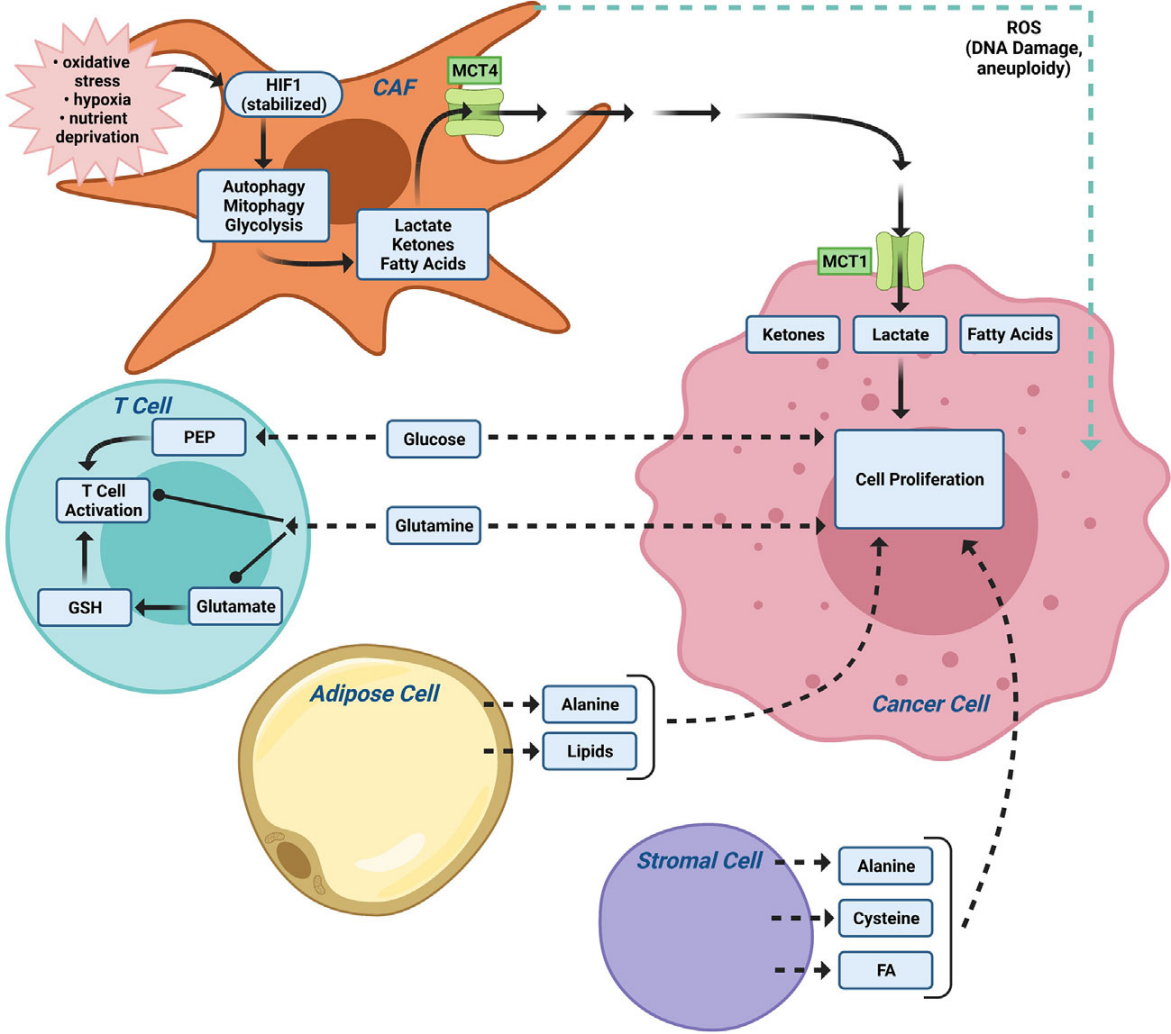
The tumor microenvironment (TME) contributes to metabolic tumor heterogeneity. In addition to CAFs (**Figure 2**), the local TME, including immune cells and adipocytes, provide nutrients for tumor metabolism. The relative importance of the different TME cellular subtypes varies between patients thereby contributing to additional levels of tumor heterogeneity. PEP (the glycolytic metabolite phosphoenolpyruvate).

Glutamine is generally required for proliferation of normal and cancer cells (115), however cancer cells have an increased demand for glutamine as a source of nitrogen. Glutamine is used for the synthesis of amino acids and nucleotides (31) (**Figure 3**). In early studies, the optimal growth of cultured HeLa cells was shown to require a 10- to a 100-fold molar excess of glutamine in culture medium compared to other amino acids (116). Furthermore, glutamine is the most rapidly used amino acid (116). The increased use of glutamine has been established in the tumor microenvironment *in vivo* (117–120) and tumors demonstrate increased uptake of ^18^F-labeled glutamine using positron emission tomography (121). Breast cancer and other cell lines may develop resistance to this metabolic dependency (122). Glutamine is required for a variety of different cellular functions in proliferating cells, providing a source of nitrogen for the synthesis of purine and pyrimidine nucleotides and for the synthesis of other amino acids (123). Glutamine is deaminated by glutaminase to generate glutamate, which is converted to α-ketoglutarate. Within the mitochondria, α-ketoglutarate is converted to oxaloacetate, citrate and malate which in turn contribute to other anabolic pathways (31) (**Figure 2**). Uptake of cellular amino acids is also affected by glutamine as intracellular glutamine exchanges with extracellular leucine *via* LAT, a plasma membrane-localized antiporter for neutral amino acids (124). The cystine/glutamate antiporter, which imports cystine to provide cysteine for protein and glutathione synthesis is also regulated by glutamate which serves as the intracellular substrate for the plasma membrane antiporter (125).

Glutamine metabolism is under physiological control by the serine-threonine kinase, mammalian target of rapamycin (mTOR) pathway. mTOR governs several important cellular functions including cellular growth, survival, protein translation and autophagy. mTOR upregulates glutaminase (GLS) thereby increasing the conversion of glutamine to glutamate. The consequent increased production of α-ketoglutarate is then used within the TCA cycle (126). Restraint of mTOR activity also increases the ability of a cell to obtain extracellular proteins as a source of amino acids (127). Induction of mTOR correlates with increased HIF and VEGF which contribute to angiogenesis. Increased mTOR activity therefore collectively stimulates glutamine uptake, glutaminolysis, glycolysis, and angiogenesis in cancer cells.

*c-myc* governs glutamine homeostasis, with direct effects and indirect actions on cellular uptake *via* transporters ASCT2 (SLC1AS) (**Figure 2**) and SN2 (SLC38A5) (128). This enhanced uptake is associated with increased conversion to α-ketoglutarate (129) and incorporation into nucleic acid synthesis (130). The dependence of cancer cells survival on glutamine has led to testing of transport inhibitors targeting ASCT2 and the glutaminase (GLS) (**Figure 2**) inhibitors, CB-839 and BPTES, for anticancer therapies (131–133).

## Heterogeneous Oxidative Metabolism in the Breast Tumor and the Tumor Microenvironment Provides Diverse Nutrients for Tumor Growth

Continued tumor growth requires the development of mechanisms to enhance access to diverse intracellular and extracellular nutrients (134, 135). Tumor cells retain a high level of metabolic plasticity, allowing them to both establish and subsequently adapt to the extracellular environment of a developing tumor. Heterogeneous tumor nutrients can be derived from tumor cells with different metabolic characteristics, in part driven by heterogeneous oxygenation within the tumor. In oxygenated tumor cells MCT1, which is expressed in breast cancers, serves as the prominent pathway for lactate uptake, which in turn serves as a substrate for tumor metabolism (47) Thus, there is a symbiosis by which glycolytic and oxidative tumor cells mutually regulate their access to energy metabolites based on heterogeneous oxygenation within the tumor (136).

### Autophagy

Autophagy provides intracellular nutrients and is upregulated in dormant breast cancer cells promoting cancer cell survival under metabolic stress (137–139). Autophagy includes, macroautophagy, microautophagy and chaperone-mediated autophagy. During macroautophagy intracellular components are enveloped in double-membraned vesicles. Lysosomes fuse with autophagososomes resulting in degradation and recycling of these substrates in the cytosol (140). The autophagic process may either enhance or retrain tumor progression depending upon the stage of tumorigenesis. In a genome-wide screen, genes that negatively regulated autophagy were also involved in cellular growth and proliferation (141). Strong evidence for an association between mitogenic signaling in the restraint of autophagy led to studies wherein the pro-mitogenic, collaborative oncogene cyclin D1, was shown to restrain autophagy in breast cancer cells by modulating the activation of AMPK (142). AMPK enhanced autophagy and in human breast cancer cells cyclin D1 restrained AMPK activity (142). Cyclin D1 reduced activation of AMPK (pT172), *via* cyclin D1-Cdk4/Cdk6 phosphorylation of LKB1, thereby inhibiting mitochondrial function and promoting glycolysis (40, 41).

### Tumor Microenvironment and Alternative Nutrients

Alternatively, the tumor microenvironment (TME) provides a rich source of distinct nutrients. Distinct cell types within the TME (cancer associated fibroblasts (CAFs), adipocytes, immune cells, tissue plasma/interstitial fluid) provide distinct nutrients to fuel tumor metabolism (**Figure 3**). Cancer-associated fibroblasts (CAFs) and adipocytes (143) can support malignant cells by providing nutrients such as alanine and lipids (143). Macrophages participate in TME metabolism (144) and MCT4 is expressed in macrophages (145). Understanding the source of nutrients for a particular tumor may provide an alternative therapeutic opportunity. Alternative substrates fueling tumor growth, include branched chain amino acids for *de novo* nucleotide and non-essential amino acid (NEAA) biosynthesis (146), acetate for acetyl-CoA and fatty acid synthesis (147), scavenging of extracellular lysophospholipids to bypass *de novo* lipogenesis (148), and macropinocytotic uptake and degradation of extracellular protein to maintain amino acid supply and bioenergetics (149, 150). Macropinocytotic uptake is induced by Ras (149, 151) and other oncogenic stimuli [reviewed in (152)]. Although the relative importance of scavenging pathways in breast cancer remains to be more fully understood, necrosis is a common feature of invasive breast cancer and breast tumor growth often outstrips the vasculature leaving tumor cells in nutrient-limited environment (153). Desmoplasia, a form of excessive fibrosis that limits perfusion, may favor the outgrowth of breast cancer cells that are capable of nutrient scavenging (154).

### Cancer Associated Fibroblasts

**Figure 4:**
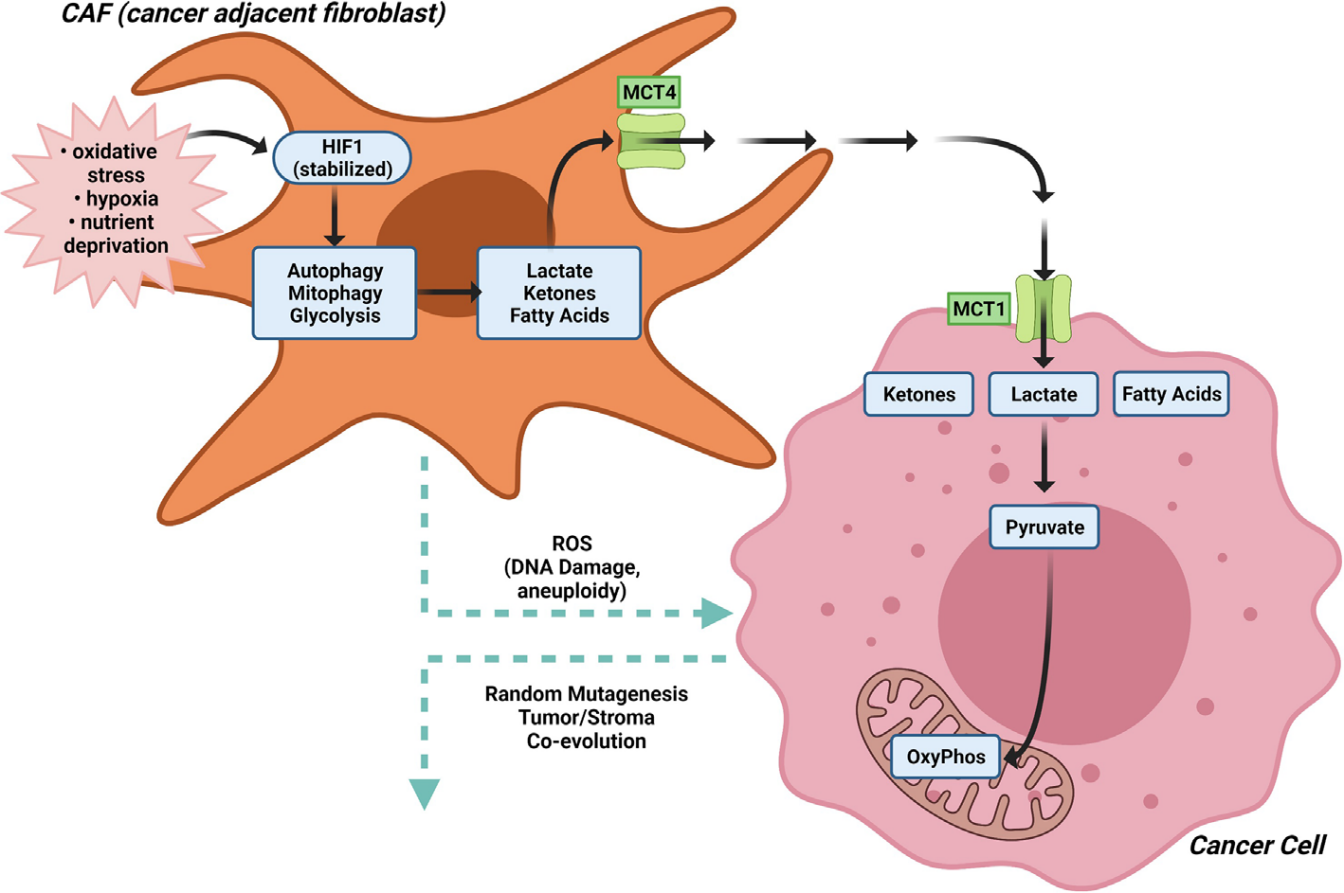
The Reverse Warburg effect. In the “Reverse Warburg Effect”, which was initially characterized in breast cancer cells, anabolic cancer cells import lactate, ketones and fatty acids released by either adjacent cancer associated fibroblasts (CAF), other stromal cell types or catabolic cancer cells, in response to oxidative stress. In CAFs, hypoxia, nutrient deprivation and oxidative stress stabilize HIF1α, which in turn causes catabolic autophagy, mitophagy and glycolysis, together with expression of the monocarboxylate transporter (MCT) 4 that exports lactate. Aerobic glycolysis in cancer associated fibroblasts (CAFs) generates energy-rich metabolites (such as lactate, ketone bodies and pyruvate) that are transferred to adjacent cancer cells, where they then enter the TCA cycle, promoting oxidative phosphorylation and increased ATP production.

The concept of scavenging alternative substrates to fuel tumor growth is illustrated by the “Reverse Warburg Effect” that was initially characterized in breast cancer cells (44, 47, 48). In the “Reverse Warburg” effect, anabolic cancer cells import lactate, ketones and fatty acids released by either adjacent cancer associated fibroblasts (CAF), other stromal cell types or catabolic cancer cells, in response to oxidative stress (21, 155). The “Autophagic Tumor Stroma Model of Cancer” proposes aerobic glycolysis in cancer associated fibroblasts (CAFs) generates energy-rich metabolites (such as lactate, ketone bodies and pyruvate) that are transferred to adjacent cancer cells, where they then enter the TCA cycle, promoting oxidative phosphorylation and increased ATP production (156–165) (**Figure 4**). In this model, hypoxia, nutrient deprivation and oxidative stress are thought to stabilize HIF1, which in turn causes catabolic autophagy, mitophagy and glycolysis, together with expression of the monocarboxylate transporter (MCT) 4 that exports lactate (21).

The molecular drivers governing the CAF metabolic phenotype may involve downregulation of caveolin-1 (Cav-1) (157). Low expression of stromal Cav-1 correlates with a high rate of tumor recurrence, metastasis, tamoxifen resistance, and poor clinical outcome in breast carcinoma (166, 167). Oxidative stress in the tumor micro-environment then activates an autophagic program, governed in part by the tumor vasculature, leading to the production of recycled nutrients that can then be used as “fuel” to promote the anabolic growth and aggressive progression of tumor epithelial cells (**Figure 3**). Autophagy in cancer-associated fibroblasts protects tumor cells against apoptotic cell death, in part through the provision of recycled nutrients. Oxidative stress in the tumor microenvironment also has mutagenic consequences (157). ROS production in cancer-associated fibroblasts, induces DNA damage and aneuploidy in adjacent epithelial cancer cells serving as a catalyst for the random mutagenesis of tumor cells and for tumor-stroma co-evolution. Bidirectional metabolic interactions are also observed with glutamine metabolism. Co-targeting glutamine synthetase in stroma and glutaminase in cancer cells reduces tumor weight, nodules, and metastasis (168).

Additional substrates participating in tumor stroma metabolic cross talk have been described in pancreatic cancer for the use of branched-chain amino acids (BCAA) (169). Pancreatic ductal cancer (PDAC)-induces branch chain amino acid transaminase 1 (BCAT1) in CAFs which govern internalization of the extracellular matrix from the tumor microenvironment to supply amino-acid precursors for branched-chain α-ketoacid (BCKA). BCKA secretion by CAF are utilized by PDCA for protein synthesis and oxidative phosphorylation (169).

### Tumor-Associated Macrophages

Tumor-associated macrophages (TAMs) and cancer cells co-exist in the context of a complex, bidirectional metabolic relationship. M1-like macrophages displaying enhanced glycolysis and reduced oxidative phosphorylation in contrast with more oxidative M2-like macrophages (170). TAMs exposed to hypoxia or lactate secrete multiple cytokines with metabolic functions, including IL6, TNF, C-C motif chemokine ligand 5 (CCL5) (112), and CCL18 (171). CCL5, and CCL18 boost the synthesis of multiple pro-glycolytic factors including HXK2, PGK1, lactate dehydrogenase A (LDHA), glucose-6-phosphate dehydrogenase (G6PD), pyruvate kinase M1/2 (PKM), pyruvate dehydrogenase kinase 1 (PDK1), pyruvate dehydrogenase (PDH), solute carrier family 2 member 1 (SLC2A1, best known as GLUT1), and vascular cell adhesion molecule 1 (VCAM1) and display lower glyceraldehyde 3-phosphate dehydrogenase (GAPDH) and succinate dehydrogenase (SDH) activity than normal macrophages. There are important consequences of tumor metabolites on immune function as Lactate secreted by glycolytic cancer cells, favors the polarization of immune cells to an immunosuppressive phenotype. Inhibiting glutamine synthetase activity in M2 macrophages skews their polarization toward an HIF1α-mediated M1 state, which impairs cytotoxic T cell recruitment and angiogenesis (172).

### Stromal Adipose Cells, Extracellular Fluids and Exosomes

Stromal adipose cells contribute to the breast tumor metabolic microenvironment. *In silico* deconvolution estimates of cell type composition and molecular profiles of constituent cell types in the context of breast tumors applied to the TCGA data revealed metabolic coupling occurs between the epithelial and stroma cell types (173). A less adipose dense stroma displayed lower levels of mitochondrial activity and were associated with tumor cells with higher levels of oxidative metabolism. An adipokine. omental cell-derived circulating ITLN1 (intelectin-1, or omentin), induced a metabolic shift in metastatic ovarian cancer cell and decrease in tumor growth rates (174). Reduced glycolysis was observed in the cancer cells *in vivo* in mice given intraperitoneally injections of ITLN1, while increased glycolysis was observed in the adjacent cancer-associated adipocytes (174).

Tissue plasma and interstitial fluid contains soluble proteins that are normally not utilized as sources of amino acids. Tumor cells may activate processes to utilize these nutrients including entosis (175), [the engulfment and degradation of entire live cells), macropinocytosis, (the bulk uptake of extracellular fluid into large vesicles (176, 177)], and micropinocytosis (178). Micropinocytosis is augmented in cancer cells, through mutations including K-Ras, and c-Src, and activation of the phosphoinositide 3-kinase (PI3 kinase) (149, 179, 180) or Hippo pathway effectors Yap and Taz (181). Pancreatic and prostate cancers bearing oncogenic mutations in *KRAS* or *PTEN*, respectively, use amino acids derived from engulfed extracellular proteins to proliferate in nutrient-limiting environment (127, 182–184).

An additional mechanism providing tumor nutrients involves CAF-derived exosomes which contain intact metabolites, including amino acids, lipids, and TCA-cycle intermediates that are avidly utilized by cancer cells for central carbon metabolism. These metabolites promote tumor growth under nutrient deprivation or nutrient stressed conditions and inhibit mitochondrial oxidative phosphorylation increasing glycolysis and glutamine-dependent reductive carboxylation in cancer cells (185).

## Altered Lipid Metabolism Within the Breast Tumor Epithelium and Tumor Microenvironment

Lipid synthesis increases in cancer cells, corresponding to an increased requirement for membrane synthesis during proliferation and cell division, cellular signaling and synthesis of hormones. Acetyl CoA carboxylase (ACC) activity is essential for breast cancer cell survival (186). Acetyl CoA carboxylase (ACC) and fatty acid synthase complex (FASN) are commonly upregulated in cancer cells (187, 188). ACC converts acetyl CoA (**Figure 3**) to malonyl CoA, rather than citrate, which in turn is converted by FAS to saturated fatty acids (SFA). Chemical inhibitors of ACC or genetic ablation of FASN by RNAi have shown some efficacy in cancer treatment (189). Lipid precursors are made available through glycolysis and through mitochondrial metabolism of glutamine through αketoglutarate to citrate (**Figure 3**). Increased glycolysis in cancer cells ensures the availability of dihydroxyacetone phosphate (DHAP) for conversion by glycerol-3-phosphate dehydrogenase 1 (GPD1) to glycerol-3-phosphate and thence phospholipids for cell membrane synthesis (190).

Expression of peroxisome proliferator-activated receptor gamma (PPAR*γ*), a key regulator of lipogenesis, is altered in breast cancer. PPAR*γ* expression is a positive prognostic factor in luminal and ductal breast cancer (191). PPAR*γ* levels are inversely correlated with tumor size, grade and TNM staging (192, 193). PPAR*γ* agonists trigger apoptosis, inhibit cell growth, decrease breast cancer cell motility and inhibit invasion of breast cancer cells (194). Ligands of PPAR*γ* inhibit the expression of several cell cycle regulators thereby reducing cancer cell proliferation (195). The synthetic PPAR*γ* ligands, rosiglitazone and troglitazone and endogenous 15dPGJ2 inhibit cyclin D1 gene expression *via* repression of *cyclin D1* transcription, leading to cell cycle arrest (196). Although the role of PPAR*γ* in tumor progression and metastasis remains controversial, in part because of the potential off target effects of PPAR*γ* ligands (197), consistent with the important role of lipogenesis in breast cancer progression, recent studies showed that genetic deletion of *Ppar*γ*1* delayed the onset of tumorigenesis by mammary epithelial cell targeted ErbB2 (198).

Recent studies have identified ferroptosis-related gene expression pathways that predict outcome in breast cancer (199). Ferroptosis is a form of regulated necrosis driven by iron-dependent peroxidation of phospholipids, plays an important role in tumor suppression (200–202). Lipid metabolism can govern ferroptosis *via* sterol regulatory element-binding protein 1 (SREBP1), a central transcription factor regulating lipid metabolism. SREBP1m targets include gene governing lipogenesis (such as ACLY, ACC, FASN and stearoyl CoA desaturase 1), gluconeogenesis and the pentose phosphate pathway (**Figure 2**) including pyruvate kinase R isoform (PKLR), phosphoenolpyruvate carboxykinase 1 (PCK1), glucose 6-phosphatase (G6PC), and glucose 6-phosphate dehydrogenase (G6PDH). Sustained activation of mechanistic target of rapamycin complex 1 (mTORC1) through oncogenic activation of the PI_3_K-AKT pathway induces SREBP1 and provides resistance to ferroptosis in breast tumors in mice (203).

## Cell Cycle Regulators Govern Tumor Metabolism

The cell-cycle governs cellular metabolism and, reciprocally, glycolytic enzyme activity can affect cellular proliferation and tumor aggressiveness, including through actions that are additional to their functions within glycolysis (204). Enhanced activity of the glycolytic enzymes, phosphoglycerate mutase (PGM) or glucose phosphate isomerase (GPI) induces proliferation of mouse embryonic fibroblasts and inhibition of these glycolytic enzymes promotes senescence (205). GPI converts glucose 6 phosphate to fructose 6 phosphate (**Figure 2**)

Regulators of cell cycle progression can also directly affect cellular metabolism. p53 for example downregulates PGM (204). Cyclin D1 overexpression restrains adipogenesis (206), suppresses mitochondrial function and biogenesis, and augments cytosolic glycolysis. The *cyclin D1* gene is overexpressed in human breast cancer and is required for oncogene-induced tumorigenesis therefore the mechanism by which cyclin D1 governs tumor metabolism is of broad interest. Cyclin D1 encodes the regulatory subunit of the holoenzyme that phosphorylates and inactivates the RB protein. Early observations in cyclin D1 anti-sense transgenic mice targeting the mammary gland showed induction of mitochondrial and lipogenic regulatory gene clusters *in vivo* (41). The induction of cyclin D1 antisense in the mammary epithelial cell of transgenic mice induced acetyl-CoA carboxylase, fatty acid synthase, hexokinase II, and pyruvate kinase (**Figure 2**). A detailed gene expression analysis evidenced the impact of increased cyclin D1 to enhance the Warburg effect (207).

Several additional mechanisms have been described by which cyclin D1 regulates cytosolic glycolysis and induces the Warburg effect. Firstly, the cyclin D1/cdk4 complex phosphorylates NRF1 at a canonical cyclin D1/CDK4 phosphorylation site. NRF1 is a key nuclear transcription factor governing mitochondrial function with targets that include mitochondrial transcription factor A (mTFA). Consequences included reduced D loop transcriptional activity in mitochondrial DNA. Deletion of the *cyclin D1* gene increased mitochondrial mass and mitochondrial activity function (40, 208). Secondly, in hepatocytes, cyclin D1–cyclin dependent kinase-4 (Cdk4) phosphorylates and activates the histone acetyltransferase, general control non-repressed protein 5 (GCN5), which then acetylates and inhibits peroxisome-proliferator-activated receptor-γ coactivator-1α (PGC-1α) activity at gluconeogenic genes (209). Thirdly, cyclin D1 increased phosphorylation of AKT^Ser 473^ in breast cancer cells and animal models, augmented AKT1 activity (210), which in turn simulates the Warburg effect (211). Collectively these studies illustrate cyclin D1 promotes the Warburg effect in tissue culture and *in vivo*.

## Mutations of Metabolic Genes in Cancer

As noted above, a number of genes and proteins that have direct roles in metabolism are regulated by oncogenes (c-Myc, cyclin D1, Ras, AKT/PI_3_K/mTOR), or tumor suppressors (p53) (212). Additionally, germ line and somatic mutations have been described in genes encoding enzymes that have direct roles in metabolism. Familial germline mutations in succinate dehydrogenase (213–215), and somatic mutations in isocitrate dehydrogenase 1 and 2 (*IDH1* and *IDH2)*, fumarate hydrase *(FH)* and isoforms of succinate dehydrogenase (*SDH)* are found in a variety of human cancers (216). IDH1 and IDH2 catalyze the decarboxylation of isocitrate to α-ketoglutarate (**Figure 2**). Fumarate hydratase catalyzes the reversible hydration of fumarate to malate. The multi-component SDH complex catalyzes the oxidation of succinate to fumarate, in concert with reducing ubiquinone in the electron transport chain. Succinate in turn may induce DNA hypermethylation (**Figure 2**). Tumors that accumulate succinate, show inhibition of 2-oxoglutarate-dependent histone and DNA demethylase enzymes, resulting in epigenetic silencing (217).

The metabolite profile itself drives oncogenesis. In the case of the *IDH1* and *IDH2* mutations, there is reduced production of αKG from isocitrate. αKG is a rate-limiting substrate for α-ketoglutarate-dependent dioxygenases that catalyze demethylation of DNA, histones and mRNA, and regulate HIF1α (212) (**Figure 2**). *IDH1* mutations in some gliomas, and *IDH1* and *IDH2* mutations in some acute myeloblastic leukemias, convert αKG to *R*-2-hydroxyglutarate (2HG). 2HG can then suppress activity in α-ketoglutarate-dependent dioxygenases through competition with αKG. Inhibition of some dioxygenases by succinate or fumarate has also been rationalized as an effector pathway for loss of function of mutations in fumarate hydratase (FH), succinate dehydrogenase (31). Fumarate derivatization of cysteine residues within the Kelch-like ECH-associated protein 1 (KEAP1) may also occur, freeing NRF2 from KEAP1-mediated degradation (218).

Recent studies of over 900 cell lines revealed diverse metabolic changes with associated potential therapeutic potential. Hypermethylation of the gene encoding asparagine synthetase showed sensitivity to L-asparaginase (219). A comprehensive proteomic analysis combined with metabalomic and gene methylation analysis revealed the metabolic heterogeneity of the cancer cell lines (220). Analysis of 225 metabolites in 928 cell lines from 20 cancer types revealed several broad principles firstly, previously described mutations (IDH1, KEAP1) revealed the predicted change in metabolites. Secondly, that common oncogenic events (*EGFR, KRAS, NRAS, TP53, PTEN, TSC1, TSC2*) had weak to non-significant associations with profiled metabolites. Thirdly, that DNA hypermethylation influence metabolite production *via* suppressing degradation pathways. For example, methylation of *SLC25A20* (carnitine/acylcarnitine translocase) in breast cancer cell lines led to accumulation of long chain acylcarnitine species. Fourthly, DNA hypermethylation regulates metabolite levels by limiting components of biosynthetic pathways. For example, hypermethylation of the *PYCR* gene, an enzyme that converts pyrroline-5-carboxylate to proline, was associated with reduced proline levels.

## Epigenetic Regulation of EMT Governs Breast Cancer Metabolism

Carcinoma cells undergo an epithelial-to-mesenchymal transition (EMT) although the transition is considered a spectrum of changes, rather than a binary event (221). EMT-inducing transcription factor (EMT-TF) regulate the induction of EMT by repressing the transcription of epithelial genes while activating mesenchymal genes. EMT-TFs are regulated at a transcriptional level by DNA methylation, histone modifications, and RNA-mediated epigenetic regulation (222). Genetic regulators of EMT also directly regulate BCa cellular metabolism (223). Many pathways link EMT-TFs expression with glycolysis, mitochondrial metabolism, glutaminolysis and lipid metabolism (224), providing the rational basis for metabolic targeting of BCa cancer EMT (223). MDA-MB-231 is a mesenchymal basal-like breast cell line with decreased mitochondrial respiration compared to the epithelial luminal-like breast cell line, MCF-7. The decrease in oxidative phosphorylation correlated with the down regulation of succinate dehydrogenase B (SDHB, complex II), the core catalytic subunit of SDH in MDA-MB-231 cells (225). Decreased SDHB expression leads to metabolic reprogramming and migration and invasion of tumor cells by promoting EMT (226–228).

Most BCa cells express both epithelial and mesenchymal traits. When epithelial cancer cells lose their epithelial features and acquire a mesenchymal phenotype this promotes motility and invasion through loss of cell polarity, disruption E-cadherin/β-catenin leading to loss of cell-cell adhesion involved in cancer invasion and metastasis (225). This E/M hybrid state is facilitated by the differential expression of Snail (Snai1 and Snai2), bHLH (Twist1 and Twist2), and zinc finger and E-box binding (Zeb1 and Zeb2), collectively termed EMT-inducing transcription factors (EMT-TFs). The mesenchymal-like phenotype is accompanied by the expression of adult stem cell programs, notably, active canonical Wnt signaling.

The EMT transition in BCa is regulated by altered expression of the transcription factors SNAIL/SLUG (229), TGF-β (230), Twist, and Goosecoid and the cell-cycle control proteins [p21^CIP1^ (231), cyclin D1 (232)]. These EMT inducing agents in turn have been shown to regulate cellular metabolism (reviewed in (233). For example, the EMT TFs Slug/Twist suppresses succinate dehydrogenase (SDH), thereby repressing mitochondrial respiration, leading to the accumulation of succinate, which suppresses TET2, causes causing DNA hypermethylation, further promoting EMT in paraganglioma (234). Recent studies identified a novel role for the cell fate determination pathway in restraining EMT. Loss of DACH1 expression, a helix-turn helix protein of the Forkhead family that is a key determinant of the cell fate determination pathway, is a predictor of metastasis and poor survival in BCa (235). The *DACH1* gene is silenced by methylation (236), and DACH1 in turns restrains the EMT program (237).

## Emerging Questions in Metabolic Heterogeneity

The increased resolution of investigative technology has provided evidence for distinct sources of metabolic heterogeneity in BCa. Metabolic heterogeneity has been identified between genetic subtypes of breast cancer and within the components of the tumor microenvironment for an individual patient’s tumor. Several key questions have emerged as a consequence of the emerging understanding that tumors are highly heterogeneous (238). How can we best harness the knowledge that genetic mutations can alter a particular patent’s tumor metabolic in order to identify therapeutic vulnerabilities?

Analysis of large compendiums of tumor cell lines has identified correlates between altered genetic changes and metabolite production (220). However, evidence suggests that most human tumors acquire hundreds of somatic mutations in coding regions (239). Taking a broader definition of a metabolic genes to include the known upstream regulators of the enzymes that actually carry out the metabolic transformation, though, reveals the extent to which mutation or altered copy number pervades human cancers (240). Metabolic gene alterations are frequent and determine tumor aggressiveness and therapy responses (219, 240). High metabolic gene abnormality frequency correlated with worse prognosis (240). The most frequent metabolic gene abnormalities in breast cancer involve lipid metabolism (240). Bystander gene deletion may also contribute to tumor metabolic heterogeneity as metabolic genes may reside in proximity to known tumor suppressor genes that are deleted in cancer. The *MTAP* (methylthioadenosine phosphorylase gene for example may be co-deleted with *CDKN2A*, resulting in elevated methyl thioadenosine which sensitizes cells to PRMT5 inhibitors (241). How then to discern the functional significance of such ubiquitous mutational loads of metabolic genes in a tumor?

Intratumoral heterogeneity subclonal driver mutations that govern tumor metabolism have been identified in breast cancer (TP53, SMAD4) (238), consistent with studies of subclonal diversification of primary breast cancer revealed by multi region sequencing of the coding region (238), the non-coding region (242) and evidence for further genetic evolution upon relapse (SWI-SNF and JAK2-STAT3) (243). What metabolic vulnerabilities emerge in tumors with metabolically diverse subclonal populations?

Mathematical modeling approaches have been developed to understand the metabolic impact of altered gene expression on tumor metabolism. Modeling analysis of epithelial-to-mesenchymal transition has been conducted, in which metabolic pathway signatures have been used to quantify the activities of glycolysis, and the citric acid cycle with corresponding analysis of enzymes governing the metabolic processes in tumor samples (233, 244). Because tumors exhibit a spectrum of EMT and a spectrum of metabolic changes which may be topologically distinct, for example in the leading *vs.* the trailing edge of an invasive tumors, more accurate mathematical predictive models are required to provide precise metabolic therapeutics.

Linking the complex patterns of metabolic genetic alterations that occurs within a tumor to therapeutic co-extinction paradigms for individualized patient treatment remains a key challenge for future research.

## Author Contributions

All authors listed have made a substantial, direct, and intellectual contribution to the work, and approved it for publication.

## Funding

This work was supported in part by NIH R01CA132115, R21CA235139-01 RP and a Breakthrough Breast Cancer Research Program grant award from Department of Defense (W81XWH1810605) RP.

## Conflict of Interest

RGP holds ownership interests in the companies ProstaGene, CytoDyn, LightSeed, Inc., and EcoGenome. RGP holds ownership interests (value unknown) of several patents and submitted patent applications.

The remaining authors declare that the research was conducted in the absence of any commercial or financial relationships that could be construed as a potential conflict of interest.

## Publisher’s Note

All claims expressed in this article are solely those of the authors and do not necessarily represent those of their affiliated organizations, or those of the publisher, the editors and the reviewers. Any product that may be evaluated in this article, or claim that may be made by its manufacturer, is not guaranteed or endorsed by the publisher.
